# Inhibition of the Growth of *Plasmodium falciparum* in Culture by Stearylamine-Phosphatidylcholine Liposomes

**DOI:** 10.1155/2011/120462

**Published:** 2011-06-14

**Authors:** Gulam Mustafa Hasan, Neha Garg, Enna Dogra, Ranu Surolia, Prahlad Chandra Ghosh

**Affiliations:** Department of Biochemistry, University of Delhi South Campus, Benito Juarez Road, New Delhi 110021, India

## Abstract

We have examined the effect of stearylamine (SA) in liposomes on the viability of *Plasmodium falciparum* in culture by studying the inhibition of incorporation of [^3^H]-hypoxanthine in the nucleic acid of parasites. Stearylamine in liposomes significantly inhibits the growth of the parasites depending on the phospholipids composition. The maximum inhibition was observed when SA was delivered through Soya phosphatidylcholine (SPC) liposomes. The chain length of alkyl group and density of SA in liposomes play a significant role in inhibiting the growth of the parasites. Incorporation of either cholesterol or Distearylphosphatidylethanolamine−Methoxy-Polyethylene glycol-2000 (DSPE-mPEG-2000) in Soya phosphatidylcholine-stearylamine (SPC-SA) liposomes improves the efficacy. Intraerythrocytic entry of intact SPC-SA liposomes into infected erythrocytes was visualized using fluorescent microscopy. No hemolysis was observed in uninfected erythrocytes, and slight hemolysis was noted in infected erythrocytes at high concentrations of SPC-SA liposomes. Overall, our data suggested SA in SPC-liposomes might have potential application in malaria chemotherapy.

## 1. Introduction

Malaria, a protozoan parasitic infection, is considered to be one of the most prevalent parasitic diseases afflicting the subtropical countries [1]. The quinoline group of antimalarial drugs such as chloroquine (CQ), mefloquine, and primaquine has been until recently, shown to be the most effective drugs for malaria chemotherapy because of their rapid onset of action and most important, their cost effectiveness, which have encouraged their wide use [2]. With the onset of CQ-resistant parasites, attempts have been made by several investigators to deliver chloroquine through liposomes for the treatment of chloroquine-resistant malaria [3–6]. The focus was also shifted either to develop novel chemotherapeutics or new modes of antimalarial drug delivery to overcome drug resistance mechanisms of the parasite [7]. Dramatic changes in the functional and structural characteristics of infected red blood cells (RBCs) are observed with *Plasmodium* infection and maturation. The alterations in lipid and protein composition-resulting from disturbance in membrane structure and function, ultimately lead to increased membrane fluidity [8]. The formation of “new permeable pathways” (NPPs) allows the entry of low molecular weight as well as nanosized molecules selectively to the parasitized RBCs [9, 10].

It has been previously reported by a number of investigators that liposomes consisting of stearylamine (SA) and phosphatidylcholine (PC) have antiprotozoan activity towards a number of pathogenic protozoan parasites like *Trypanosoma cruzi *[11]*, T. brucei gambiense *[12]*, Toxoplasma gondii *[13], and *Leishmania donovani *[14]. The aim of this study was to evaluate the antimalarial effect of SA-intercalated PC liposomes on infected erythrocytes in culture. The effect of various liposomal components/constituents like phospholipids, the density of cholesterol, density and chain length of alkyl group of SA, and DSPE-mPEG-2000 which are known to influence significantly the efficacy of liposomal drugs, was coherently studied. Fluorescent analogue of SA (octadecylrhodamine) was used both as a marker for SA intercalation as well as a tracking molecule to visualize the entry of SPC-SA (Soya phosphatidylcholine-Stearylamine) liposome into infected RBC.

## 2. Methods

### 2.1. Chemicals

All chemicals were obtained from Sigma Chemical Co., St. Louis, Mo, USA or GIBCO BRL, Gaithersburg, Md, USA unless otherwise indicated. Soya Phosphatidylcholine (SPC) was obtained as a gift from Lifecare Innovations Pvt. Ltd., Gurgoan, India. DSPE-mPEG-2000 (Distearoyl phosphatidyl ethanolamine-Methoxy-Polyethy-lene glycol-2000) was purchased from Avanti Polar Lipids, Inc., Alabaster, Ala, USA. Hypoxanthine Monohydrochloride, [^3^H(G)] was bought from American Radiolabeled Chemicals, Inc., St. Louis, Mo, USA. Human O^+^ serum was obtained from Innovative Research, Inc., Peary Court Novi, Mich, USA. 

A fluorescent amine analogue, octadecylrhodamine was purchased from Invitrogen Corp., Grand Island, NY, USA. The stock solution was prepared by dissolving the compound at 1.25 mM in ethanol and was stored at −20°C protected from light.

### 2.2. *In Vitro* Culture of *Plasmodium falciparum*

The strain of *Plasmodium falciparum* used in the study was 3D7 obtained from National Institute of Malarial Research, New Delhi, India. This strain was maintained (a modified method of Trager and Jensen [15]) by serial passages in human erythrocytes cultured at 4% hematocrit in RPMI-1640 media supplemented with 10% human serum and incubated at 37°C under the atmosphere of mixed gas (containing 5% CO_2_, 5% O_2_, and 90% N_2_) in a plastic chamber. Heparinized whole O^+^ blood was collected from Rotary Blood Bank, New Delhi, and RBCs were separated under sterile conditions by centrifugation to remove serum and buffy coat. The levels of parasitemia were routinely monitored on blood smear with 5% Giemsa azure type B stain in phosphate buffer (20 mM, pH 7.2). For each experiment, samples of the stock culture were further diluted in culture medium up to 2% hematocrit and parasitemia of 1% in preparation for the addition to microtitration plates. 

### 2.3. Liposomes Preparation

**Figure 1:**
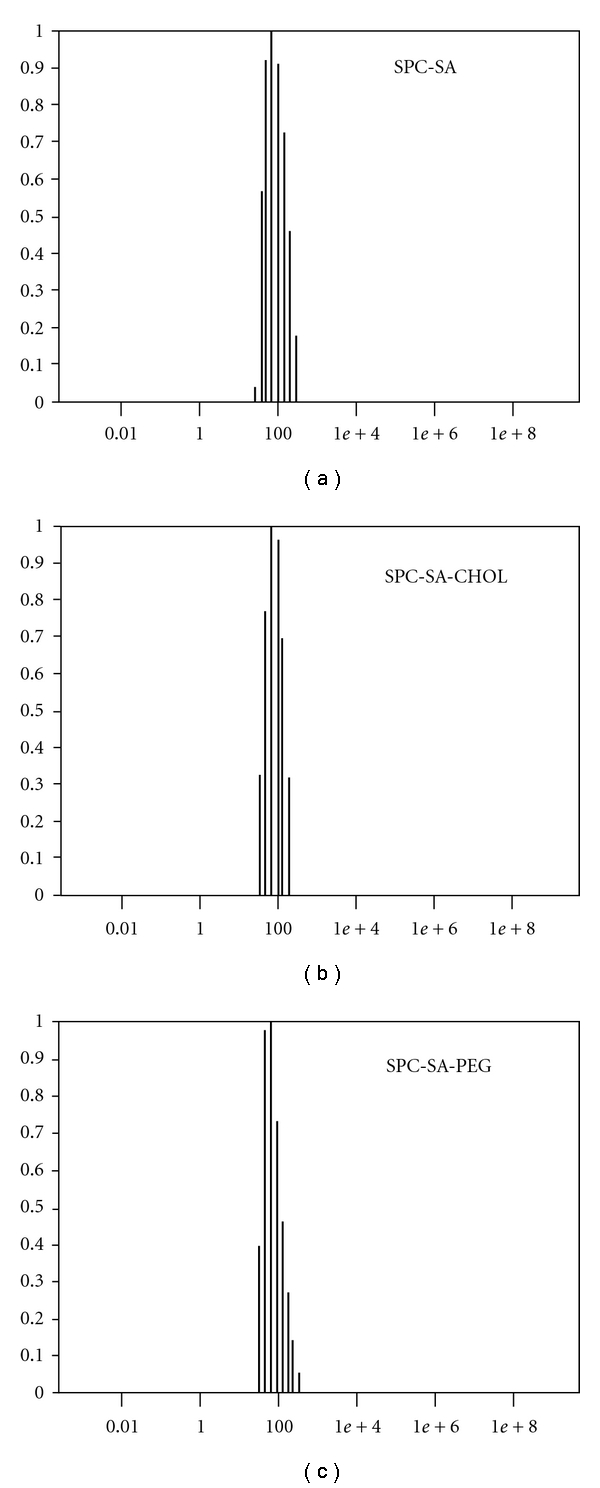
Particle size determination of SPC-SA liposomes using Malvern Zetasizer: Mean diameter of the liposomes was found to be (a) 100 nm for SPC-SA, (b) 120 nm for SPC-SA-CHOL (CHOL: Cholesterol, 20 mol%), and (c) 90 nm for SPC-SA-PEG (PEG, 5 mol%). *x*-axis denotes diameter of liposomes (in nm) and *y*-axis Intensity (%).

Liposomes were prepared by the hand-shak method as described earlier [16]. Briefly, SPC or EPC (Egg phosphatidylcholine) alone or with either SA or PA (Phosphatidic acid) in a molar ratio of 80 : 20 (40 *μ*mole total lipids) and trace amount of octadecylrhodamine (R_18_) (41 nmoles/40 nmoles total lipid), as a marker for estimation of SA intercalation, was dissolved in chloroform (2-3 mL) in 100 mL round-bottom flask. The chloroform was evaporated to dryness at 30°C, under reduced pressure, by using rotary evaporator. The thin film, so formed, was desiccated for 1h, followed by hydration with 1ml phosphate buffer saline (PBS 20 mM, pH 7.2) for overnight at 4°C. On the following day, liposomes were sonicated at 25°C for 30 min in a bath-type sonicator (Branson 1510) at 40 kHz and extruded through 100 nm polycarbonate membrane. The liposomal SA was separated from free SA by ultracentrifugation (Beckman coulter ultracentrifuge, 300,000× g, 40 min, 4°C). The pellet was suspended in 1ml PBS (20 mM, pH 7.2) and finally filtered through 0.22 *μ*m Millex GV Millipore membrane. Liposomes containing stearylalcohol, stearic acid and different chain length fatty acids were prepared as described above using molar ratio of SPC : other components 80 : 20. The recovery of liposomes was monitored by measuring phospholipid using Stewart's method [17] and SA was assessed fluorimetrically (excitation 560 nm/emission 590 nm). Recovery of liposomes was found to be 60–65% with 90–95% SA intercalated in all the liposomal formulations. Stearically stabilized liposomes were prepared as described above by adding various mol% (1–5 mol%) of DSPE-mPEG-2000 during the preparation of lipid film. The size of the liposomes was measured using Malvern Zetasizer, Nano ZS model, and it was found in the range of 90–110 nm (Figure 1). For the preparation of fluorescent labeled-liposomes for fluorescence microscopy, octadecylrhodamine (R_18_) (6 nmole/200 nmoles total lipid) was added in SA-SPC liposome suspension and incubated at room temperature for 15 min. Excess R_18_ was removed by ultracentrifugation (300,000× g, 40 min, 4°C), and the pellet obtained was resuspended in 20 mM PBS (final concentration 1 mg/mL of phospholipid). 

### 2.4. Evaluation of Liposomal Antimalarial Activity

Antimalarial effects of the different liposomal formulations was monitored by studying the inhibition of incorporation of [^3^H]-hypoxanthine in the nucleic acid of parasites [18]. In brief, liposomal preparations were serially diluted and added to *Plasmodium falciparum* infected erythrocyte suspension (2% final hematocrit and 1% parasitemia) in a 96-well tissue culture plate. After 24 h of incubation at 37°C, 0.2 *μ*Ci of [^3^H]-hypoxanthine was added to each well and cells were harvested 18 h later by using a Skatron Semiautomated cell harvester. [^3^H]-hypoxanthine incorporation in nucleic acid was measured in a liquid scintillation counter (Model: Tri-Carb 2900 TR, Perkin Elmer), and inhibition of growth was calculated by comparison with control (Control consists of complete medium in a substitute for the test molecule.) All data points were collected in triplicate for each experiment. The IC_50_ (concentration of SA required to inhibit 50% of growth of parasites as measured by incorporation of [^3^H]-hypoxanthine) of the data was generated using Origin Cal software (version 3).

### 2.5. Intracellular Accumulation of SA-Liposomes

The interaction of SA liposomes with infected erythrocytes was visualized by performing a time-dependent study using octadecylrhodamine (R_18_)-SPC-SA liposomes. The erythrocytes cultured at 2% hematocrit and high parasitemia (8–10%) were exposed to labeled liposomes at its IC_50_ for 15, 30, 45, and 120 min and kept at 37°C under the mixed gas atmosphere as mentioned previously. At the end of incubation, unbound liposomes were washed off by brief spin and cells were fixed with a mixture of 2% paraformaldehyde and 2.5% glutaraldehyde for 2h in 0.1 M PBS at 4°C. Staining of parasites was done by using DAPI stain (1 *μ*g/mL) for 30 min at 37°C and after subsequent wash cells were visualized on a fluorescent microscope (Nikon Eclipse TE 2000) using rhodamine filter 96312/G2EC (excitation 540–525 nm/emission 620–660 nm) and DAPI filter (excitation 359 nm/emission 461 nm). Uninfected erythrocytes were taken as control.

### 2.6. Assessment of Hemolytic Activity

Hemolysis was measured in SPC-SA-liposome-treated sets of cultured infected and uninfected erythrocytes by measuring absorbance of Hb at 405 nm (soret peak of hemoglobin (Hb)) as described [19]. Briefly, increasing concentration of liposomal preparations was added in *Plasmodium falciparum* infected (2% hematocrit and 1% parasitemia) and uninfected erythrocyte (2% hematocrit) in a 96-well plate for 42 h at 37°C. After incubation, the plate was spun down briefly and absorbance of supernatant was measured at 405 nm. Mixing the erythrocytes with 1% of Triton-X 100 attained the complete hemolysis. Cells in PBS were taken as negative control.

## 3. Results

### 3.1. Effects of Various Liposomes on the Viability of *Plasmodium falciparum  In Vitro*

The effect of various liposomes on the viability of *Plasmodium falciparum* strain 3D7 in culture was tested (Table 1). SPC liposome alone or having negatively charged lipid, phosphatidic acid (PA), had no effect on the viability of parasites. However, SPC liposomes having positively charged lipid, stearylamine (SA), significantly inhibited the growth of the parasites (IC_50_ = 6.87 *μ*M (Figure 3) & 24.08 *μ*M in terms of phospholipid in Table 1). To further dissect the role of alkyl and amino group of SA in plasmocidal activity, SPC liposomes containing either stearyl alcohol or stearic acid having no amine group were prepared and tested. Effect of spermidine—a polyamine having multiple amino groups and no alkyl group—was also examined. Stearyl moiety alone as in stearic alcohol and stearic acid did not show any effect as concluded by the lack of antimalarial activity. Even multiple amino groups as in spermidine had no effect on growth of parasites (Table 1). Different chain length fatty acids and alkyl alcohols ranging from tetradecyl to octadecyl (stearyl) did not show any inhibitory effect (Table 1). These results clearly suggest that both alkyl and amino groups of SA are essential in the inhibition of growth of *Plasmodium falciparum* in culture as stearylalcohol without amino group and spermidine without alkyl group but with three amino groups have no antimalarial activity.

### 3.2. SA Density in Liposome Membrane Dictates Plasmocidal Activity

**Figure 2:**
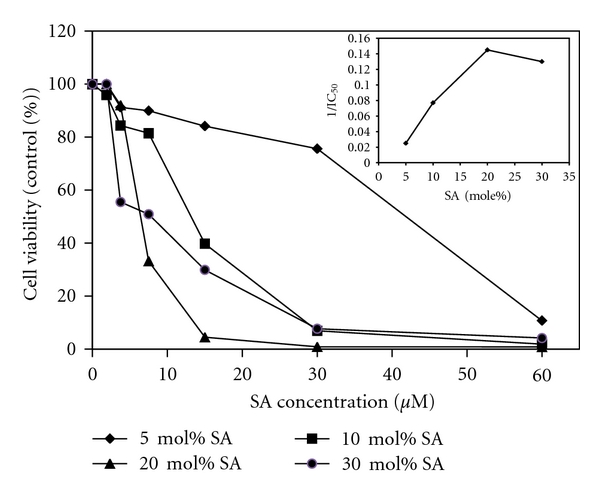
Effect of SA density in SPC liposome on the parasite viability: Infected human RBCs in the presence of liposome suspension (30 *μ*M) containing 5, 10, 20, and 30 mol% SA was incubated at 37°C for 42 h as described in materials and methods. Inset: Relationship between surface density of SA in the liposome membrane and reciprocal of the IC_50_ required for killing the parasite (1/ IC_50_).

After incubating the cells with liposomes loaded with various mol% of SA, a linear increase in antimalarial activity was observed till 20 mol% (IC_50 _ = 6.87 *μ*M) after which there was no further enhancement. The liposomes consisting of 5 mol% SA showed very weak antimalarial activity (IC_50_ = 41.15 *μ*M) (Figure 2). This indicates that the optimum mol% of SA in liposomes to confer maximum killing activity was 20 mol%. All subsequent experiments were carried out using 20 mole% SA in liposomes.

### 3.3. Role of Chain Length of Alkyl Amine on the Inhibition of Growth of *Plasmodium falciparum* in Culture

*Plasmodium falciparum* infected red blood cells suspension was incubated with liposomes composed of 80% soya PC and 20% alkyl amines of different chain length, and their viability was examined. The results depicted in Table II suggested that the chain length of the alkyl group of the aliphatic amines is directly proportional to antimalarial activity. The liposome containing decylamine has IC_50_ of 43.45 *μ*M compared with that of octadecylamine (SA) having IC_50_ of 6.87 *μ*M (Table 2) suggesting that the hydrophobic portion of alkyl amine plays a synergistic role along with the amino group in plasmocidal activity.

### 3.4. Antimalarial Activity of SA Liposome Is Phospholipid Dependent

**Figure 3:**
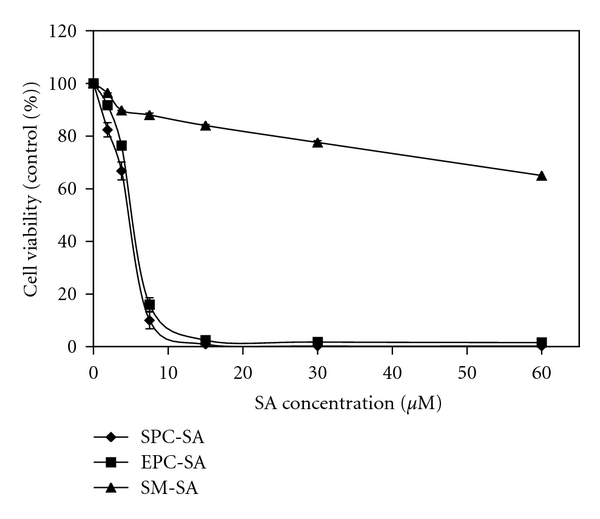
Effect of different composition of liposome on plasmocidal activity of SA: SPC, EPC, and sphingomyelin liposomes containing 20 mol% of SA were added in the cultured cells as described in materials and methods. A graph of cell viability (measured after 42 h) against SA concentration provides the IC_50_.

The phospholipid is the main constituent of liposomes and phospholipid composition of liposomes significantly influence the biological efficacy of encapsulated drugs [20]. In order to ascertain whether incorporation of SA in different phospholipids has any effect on the inhibition of growth of *Plasmodium falciparum* in human erythrocytes in culture, we have incorporated SA in three different phospholipids like bovine brain sphingomyelin, egg phosphatidylcholine, and soya phosphatidylcholine and studied its effect on the anti-malarial efficacy of SA in culture. SA in SPC and EPC liposomes with IC_50_ of 6.87 *μ*M and 7.30 *μ*M, respectively, was equally effective as an anti-malarial agent (Figure 3). On the other hand, it had no effect when incorporated in sphingomyelin liposomes; clearly showing that anti-malarial efficacy of SA in liposomes is significantly dependent on the phospholipid composition of liposomes.

### 3.5. Incorporation of Stabilizers in SPC-SA Liposomes

**Figure 4:**
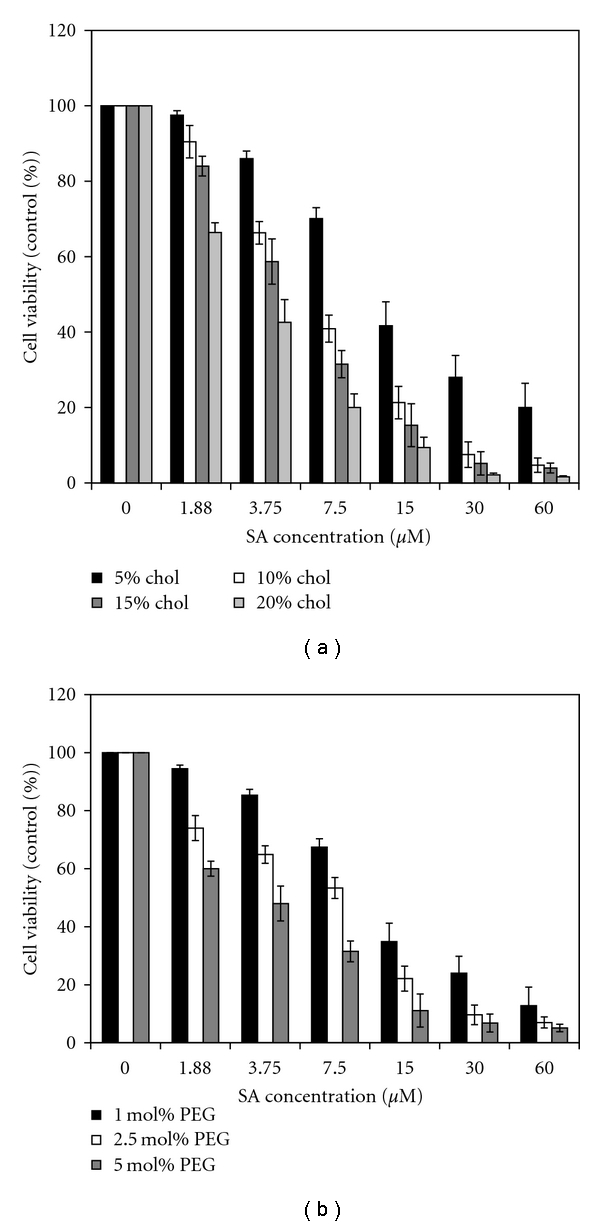
Effect of density of cholesterol or DSPE-mPEG-2000 in SPC-SA liposome on plasmocidal activity: cells (1% parasitemia and 2% hematocrit) in the presence of increasing concentrations (1.88 *μ*M–60 *μ*M SA) of SPC-SA liposome containing different density of (a) cholesterol (5, 10, 15, and 20 mol%) (b) DSPE-mPEG-2000 (1, 1.25, and 5 mol %) cultured for 42 h at 37°C. The results are expressed as a percentage of the incorporation of the radioactive precursors as compared to controls incubated in the absence of any liposome.

It is well known that cholesterol increases the stability of liposomes in the physiological environment, and DSPE-mPEG-2000 increases the hydrophilicity on the surface of liposomes, consequently increasing the longevity of liposomes in the blood circulation and improving the therapeutic efficacy of a number of drugs [21, 22]. In our case, incorporating cholesterol and DSPE-mPEG-2000 in SA liposomes marginally increased the efficacy (Figures 4(a) and 4(b)) suggesting that these liposomes can be used as a delivery vehicle for SA for the treatment of malaria *in vivo* condition. 

### 3.6. Direct Entry of SPC-SA Liposomes Specifically into Infected Erythrocytes

**Figure 5:**
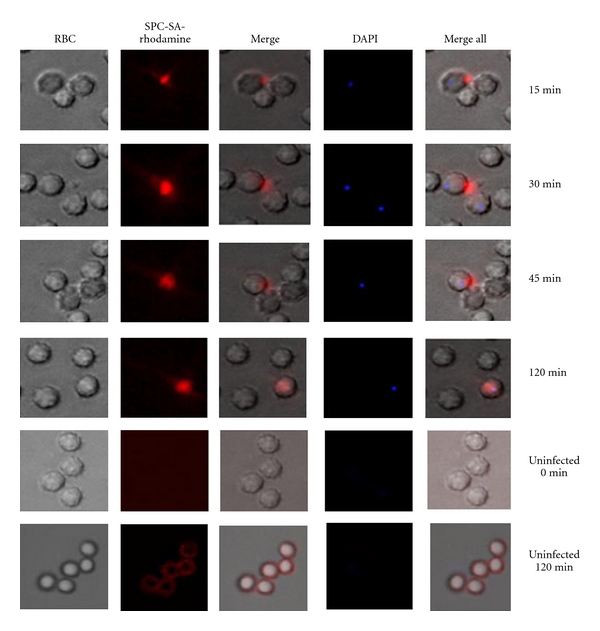
Intraerythrocytic uptake of SPC-SA liposome: parasite cultures were exposed for different time points with rhodaminated SPC-SA liposomes (5 *μ*g/mL) (Panel: SPC-SA Rhodamine) at 37°C, fixed and further counterstained using DAPI (1 *μ*g/mL), and kept in dark. Images were taken within 5 min after exposure to microscopic light to avoid photo bleaching. Uninfected erythrocytes were also treated under similar conditions. Panel RBC denotes phase contrast pictures of RBC, Merge denotes merger of phase contrast picture and rhodamine picture.

A time-dependent study was performed to probe the initiation of the interaction of SA liposome with infected RBCs. After 45 minutes exposure to the SPC-SA liposome, internalization of the liposome into infected RBCs began, which became completely evident after 2 h of incubation (Figure 5), indicating that SPC-SA liposome was able to find its way through the host cell membrane. However, uninfected erythrocytes showed the accumulation of liposomes at their surface even after 2 h of incubation clearly suggesting that the entry of SPC-SA liposome is moreover restricted to parasitized erythrocytes only.

### 3.7. Plasmocidal Activity of SPC-SA Liposome without Hemolysis

To probe the mechanism of antimalarial activity of SA liposome, the ability to induce hemolysis of infected cells was investigated. No hemolysis was observed in uninfected erythrocytes. However, in infected erythrocytes at a higher dose (60–1.88 *μ*M), hemolysis of about 5% was observed with the effect subsiding at the IC_50_ value of SPC-SA liposome (Table 3), suggesting that the antimalarial effect of these liposomes is independent of hemolysis.

## 4. Discussion

Several studies have reported that SA in liposomes kills a number of pathogenic protozoan parasites in culture as well as in animal model [11, 13, 14, 23, 24]. The results of the current study showed for the first time the inhibition of growth and multiplication of *Plasmodium falciparum* by these liposomes. It has been reported that the interaction of liposomes with mammalian cells is significantly dependent on the composition, charge, size, rigidity, and hydrophilicity on the surface of the liposomes as well as the cell types [25]. Therefore, to examine the role of these parameters in the antimalarial activities of SA liposomes, modifiers were incorporated in SPC-SA liposomes. The maximum inhibition was observed when SA was delivered through soya-PC followed by egg-PC liposomes with no inhibition when SA was delivered through SM liposomes. Alone egg-PC or soya-PC liposomes or SPC-PA liposomes were unable to elicit any plasmocidal activity.

As stated earlier, phospholipid composition plays a significant role in determining the biological efficacy of encapsulated drugs in liposomes. It is known that sphingomyelin (SM) forms very rigid liposomes as compared to liposomes prepared by EPC or SPC. It has also been reported that fusion of liposomes with mammalian cells [26], transfer of individual lipid molecules from liposomes to the plasma membrane of the mammalian cells or lipoproteins [27, 28] and endocytosis of liposomes [29] are significantly dependent on the lipid composition and fluidity of the liposomes. The reduced efficacy of SA in SM liposomes in inhibiting the growth of *Plasmodium falciparum* as compared to EPC or SPC liposomes may be due to reduced fusion/interaction of rigid SM liposomes with infected erythrocytes as compared to fluid EPC or SPC liposomes.

Increasing the surface density of SA in the liposomal membrane resulted in a progressive increase in the inhibition of growth of parasites, and liposomes, containing <5 mol% were possibly inactive although SA has been 90–95% intercalated in all the liposomal formulations. The explanation for this observation has been attributed to polycationic nature of SA by earlier investigators [13]. However, there has been no report in the literature about the dependency of the hydrophobic domain of SA. In order to answer this question, liposomes composed of 80% soya PC and 20% alkylamines of different chain length were prepared, and their plasmocidal activity was examined. It was found that increasing the chain length of the aliphatic amines increases antimalarial activity (Table 2) suggesting that not only polycationic surface but also the hydrophobic portion of the alkyl amines is very important and involved in the antimalarial activity. This may explain why polycationic molecules, like spermidine, polylysine, polyhistidine, and polymyxin B that lacks hydrophobic domain, are unable to kill parasites. 

The mechanism by which SPC-SA liposomes kill malaria parasite is not well understood at present. But reports suggest that these liposomes kill other protozoan parasites by direct interaction with the negatively charged surface molecules of the parasites [24]. Now, question is how malaria parasite infected mature red blood cell, which is a terminally differentiated cell and lacks endocytic activity, internalized positively charged SA incorporated into SPC liposomes? Infection of human erythrocytes by the malarial parasites, *Plasmodium falciparum*, results in complex membrane sorting and signaling events in the mature erythrocytes [30]. Host-signaling components in combination with parasite protein makes the intraerythrocytic environment conducive to induce formation of the membrane surrounding malaria vacuole [31]. Erythrocytes membrane contain detergent-resistant membrane (DRM) raft, which contains many proteins, and some of these proteins (minor DRM proteins) are enriched in the parasitophorous vacuolar membrane formed by the malaria parasite as it enter the erythrocytes [32]. These results suggest that entry of erythrocyte membrane protein into the cytosol occurs through membrane invagination in malaria-infected erythrocytes. It has also been reported that once inside the erythrocytes, malaria parasite remodels the erythrocyte membrane creating a number of transport pathways for importing nutrient molecules from the circulation rendering it more permissive for malaria parasite survival [33–35]. Reports suggested that channels called “New Permeability Pathways” or NPPs appear after 12–16 h of *Plasmodium* invasion, and small molecules (MW < 200 Da) gained access to the parasite through it [9, 10]. It has also been reported that parasite has direct access to extracellular nanosized (80 nm) latex bead particles[36]. To understand the mechanism of internalization of SPC-SA liposomes, we have tracked entry of octadecylrhodamine-SA liposomes (having size 90–110 nm) into infected RBCs by fluorescence microcopy. It was found that octadecyl rhodamine probe enters into the cytosol of infected erythrocytes (Figure 5). However, the exact mechanism of entry of the probe into cytosol and from cytosol of infected erythrocytes into parasitophorous vacuole is not well understood. 

The spontaneous transfer of the probe octadecyl rhodamine to unlabelled membranes has been reported [37]. Further, Grellier et al. have demonstrated the existence of the unidirectional flux of lipid from HDL to the intracellular parasites via the erythrocyte plasma membrane without internalization of the lipoprotein particles [38]. They have also demonstrated that the cytoplasm of infected erythrocytes contains numerous vesicles and tubular structure. Tubular structures acts as connector between parasitophorous vacuolar membrane system and the erythrocytes membrane for transport of lipids. Our results suggest that both SPC and SA were transferred to the membrane of *Plasmodium-*infected erythrocytes following fusion between SPC-SA liposomes and infected erythrocytes. Then, SA was transported from the infected erythrocytes membrane to the parasites possibly via lateral diffusion followed by fusion with complex network of membranes occurring in the cytosol of *Plasmodium*-infected erythrocytes as reported by Grellier et al. [38].This may explain the labeling of infected erythrocytes with octadecyl rhodamine probe. Besides, various reports suggested that parasites take up hemoglobin from cytosol of RBC by plethora of pathways. A well-characterized vesicular pathway for internalization of host cytoplasm is via cytostome-derived invagination resembling endocytosis [39, 40]. Recently, Elliott et al. [41] showed that four different but overlapping pathways exist in *Plasmodium falciparum *for taking up hemoglobin from cytosol of RBC. They have shown that early ring-stage parasites undergoes profound morphological changes in which they fold like a cup onto themselves and during this process (termed as “Big Gulp”) engulfs a large amount of cytosol. In addition, different processes like endocytosis via cytostome, cytosomal tubes arising from cytostome and phagotrophy resembling phagocytosis occur at later stages [41]. A cytostome-mediated but vesicle-independent process model of hemoglobin uptake in *Plasmodium falciparum* has also been demonstrated [42]. Therefore, it is tempting to speculate that parasite use either one or all of the above pathways for taking membrane bound SA from cytosol. The interaction of SA with the malaria parasite in the parasitophorous vacuoles in erythrocytes may be facilitated by its high negative surface charge [43]. 

In the present study, it is clearly demonstrated for the first time that SA incorporated into SPC liposomes enter into malaria infected RBCs and are very effective in the inhibition of the growth and multiplication of the *Plasmodium falciparum* within erythrocytes. The low toxicity of these liposomes (Table 3) to the host erythrocytes may find SA-bearing liposomes an effective means of chemotherapy and control of *Plasmodium falciparum* infection in human.

##  Authors Contribution

G. H. Mustafa and N. Garg contributed equally to the paper.

##  Conflict of Interests

The authors report no conflict of interests.

## Figures and Tables

**Table 1:** Effects of Various Liposomes on the Viability of *Plasmodium falciparum in vitro. *

Types of liposomes	IC_50_ in terms of phospholipid concentration (*μ*M)
SPC	>208.33
SPC: PA	>208.33
SPC: SA	24.08 ± 1.15
SPC: Stearic acid (octadecanoic acid)	>208.33
SPC: Stearyl alcohol (octadecyl alcohol)	>208.33
SPC: myristic acid ( tetradecanoic acid)	>208.33
SPC: Palmitic acid (hexadecanoic acid)	>208.33
SPC: myristyl alcohol (tetradecyl alcohol)	>208.33
SPC: Cetyl alcohol (hexadecyl alcohol)	>208.33
Free spermidine	>1102

Cells with 1% parasitemia and 2% hematocrit in the presence of various concentrations of above mentioned liposomal formulations (6.5 *μ*M–208 *μ*M lipids) and free spermidine (34 *μ*M–1102 *μ*M) were treated for 42 h at 37°C (in between at 24 h, [^3^H] hypoxanthine was added). The cell-associated radioactivity was determined and inhibition of growth calculated by comparison with controls (without liposomes/spermidine).

**Table 2:** Effect of different chain length alkylamine in SPC liposome on parasite growth after 42 h in culture.

Types of liposomes	IC_50_ (*μ*M)
SPC: Decylamine CH_3_(CH_2_)_9_NH_2_	43.45 ± 4.45
SPC: Dodecylamine CH_3_(CH_2_)_11_NH_2_	24.65 ± 2.69
SPC: Tetradecylamine CH_3_(CH_2_)_13_NH_2_	13.59 ± 1.64
SPC: Hexadecylamine CH_3_(CH_2_)_15_NH_2_	10.44 ± 1.20
SPC: Octadecylamine CH_3_(CH_2_)_17_NH_2_	6.87 ± 0.44

Parasites were treated with indicated alkylamine SPC liposome for 42 h with concentration range of (1.88 *μ*M–60 *μ*M alkylamine). IC_50_ value was assessed by measuring [^3^H] hypoxanthine incorporation as described in Materials and Methods. The mean value ± standard deviation is indicated for each group, and values are representative of 3 separate experiments.

**Table 3:** Effect of various concentrations of SPC liposomes containing 20 mol% SA on the hemolysis of infected & uninfected erythrocytes.

Concentration of liposomes (SA) *μ*g/mL	% Hemolysis	
	Infected RBC (1% parasitemia)	Uninfected RBC
16.0	3.30 ± 0.34	0
8.0	1.91 ± 0.62	0
4.0	1.59 ± 0.85	0
2.0	0	0
1.0	0	0
0.5	0	0

SPC-SA liposomes at different concentrations were mixed with infected (1% parasitemia) and uninfected erythrocytes for 42 h as described in materials and methods. Percentage hemolysis was calculated using the expression % hemolysis = [A_405 nm_ (sample) − A_405 nm_ (negative control)]/A_405 nm_ (positive control).
